# Effect of Fermented Corn-Soybean Meal on Serum Immunity, the Expression of Genes Related to Gut Immunity, Gut Microbiota, and Bacterial Metabolites in Grower-Finisher Pigs

**DOI:** 10.3389/fmicb.2019.02620

**Published:** 2019-11-20

**Authors:** Junfeng Lu, Xiaoyu Zhang, Yihao Liu, Haigang Cao, Qichun Han, Baocai Xie, Lujie Fan, Xiao Li, Jianhong Hu, Gongshe Yang, Xin’e Shi

**Affiliations:** Key Laboratory of Animal Genetics, Breeding and Reproduction of Shaanxi Province, Laboratory of Animal Fat Deposition and Muscle Development, College of Animal Science and Technology, Northwest A&F University, Yangling, China

**Keywords:** fermented feed, corn-soybean meal, immunity, microbiota, metabolite, pig

## Abstract

Fermented corn-soybean meal (fermented feed, FF) is commonly used in swine production, but the effects of FF on gut health remain unclear. In this study, serum immunity, mRNA abundances of antimicrobial peptides (AMPs) and Toll-like receptors *(TLR1-9*), bacterial abundance in the duodenum and colon, and colonic metabolic phenotypes were determined in crossbred barrows (Duroc × Landrace × Large White) fed FF or normal feed (unfermented feed, UF) (*n* = 6). When compared to the UF group, the results showed that serum levels of IgG and IgM were significantly increased in FF group pigs (*P* < 0.05). FF significantly decreased the abundances of Bacteroides and Verrucomicrobia in the duodenum and decreased the abundances of Bacteroides, Proteobacteria, and Verrucomicrobia in the colon while it significantly increased the abundances of Firmicutes and Actinobacteria (*P* < 0.05). Furthermore, a Spearman’s correlation analysis showed that serum immunity and the expression of genes related to gut immunity were associated with bacterial strains at the family level. Moreover, differentially abundant colonic microbiota were associated with colonic metabolites. LC-MS data analyses identified a total of 1,351 metabolites that markedly differed between the UF and FF groups. C5-Branched dibasic acid metabolism was significantly upregulated whereas the purine metabolism was significantly downregulated (*P* < 0.05) in the colonic digesta of pigs in the FF meal group compared to the UF meal group. Collectively, these results indicated that FF meal could influence serum immunity and the expression of genes related to gut immunity, correlating with the gut microbiota and bacterial metabolites in grower-finisher pigs. This study may provide an alternative strategy for improving the intestinal health of grower-finisher pigs.

## Introduction

Antibiotics have long been used in swine production to maintain health and productivity. In recent years, the overuse of antibiotics in swine production has become an important concern (van der Fels-Klerx et al., 2011) and has led to the development of alternatives to antibiotics in feeds (Dibner and Richards, 2005), such as probiotics, prebiotics, and organic acids, for use in swine husbandry (Thacker, 2013; Markowiak and Slizewska, 2018). The microbial fermentation of feed has been proposed as a potential alternatives (Verstegen and Williams, 2002).

Extensive evidence has shown that microbial fermentation can improve the nutritional quality of pig feed by increasing the bioavailability of nutrients (Borling Welin et al., 2015; Jeong et al., 2016) and reducing the content of anti-nutritional factors (Missotten et al., 2015; Shi et al., 2017; Wang et al., 2018). Moreover, the use of fermented corn-soybean meal has been shown to improve the immune function of pigs (Zhu et al., 2017), as the consumption of FF has a positive impact on the gut microbiota and can improve the fecal microbial count and intestinal morphology in grower-finisher pigs (Dowarah et al., 2017). However, so far, information on the effects of FF on the health of pigs is limited.

Many studies have highlighted the correlation between gut microbiota and intestinal morphology, regulation of immunity, digestion of carbohydrates, and body health in livestock (Richards et al., 2005; Stanley et al., 2016; Gu et al., 2019; Guevarra et al., 2019). Recent evidence suggests that the gut microbiota plays a crucial role in livestock health and disease (Tremaroli and Backhed, 2012). A growing number of studies indicate that the homeostasis and composition of the gut microbiota is dynamically formed by many factors (Ananthakrishnan, 2015; Zhao et al., 2015), including age, time, feed, and probiotics (Isaacson and Kim, 2012). For instance, in grower-finisher pigs, fermented Mao-tai lees modulate the gut microbiota and increase the abundance of the potentially beneficial bacteria Lactobacillus and Akkermansia (Wang J. et al., 2019). Moreover, 45% feed of fish meal can be replaced by fermented soybean meal without negative effects on growth performance and intestinal integrity of juvenile large yellow croaker (Wang P. et al., 2019).

It remains uncertain whether FF boosts immunity and the composition of the gut microbiota of swine. Therefore, the aim of the present study was to assess the effects of corn-soybean FF on serum immunity factors and the expression of genes related to gut immunity. The results of an intestinal microbiota analysis indicated that corn-soybean FF significantly changed the composition of the gut microbiota and that metabolites significantly differed between pigs fed FF meal versus UF meal.

## Materials and Methods

### Ethics Statement

This study was carried out in accordance with the recommendations of the Animal Welfare Committee of Northwest A&F University (Yangling, Shaanxi Province, China). The protocol was approved by the Animal Welfare Committee of Northwest A&F University.

### Preparation and Composition of FF

Corn-soybean meal was purchased from Beijing Dabeinong Technology Group Co., Ltd. (Beijing, China). Effective Microorganisms^TM^ used in the present study are a mixture containing 60% *Lactobacillus*, 20% *Clostridium*, and 8% *Bifidobacteria* and were purchased from Nongfukang Biological Technology Co., Ltd. (Zhengzhou, China) and diluted 1:30 (w/v) with sterile water. In accordance with the manufacturer’s instructions, the corn-soybean meal was mixed with probiotics and incubated at 27–32°C for 36 h. We then determined the live bacteria in the fermented product, and the final number of microorganisms was guaranteed at a concentration of 2 × 10^9^ CFU/g. The method for CFU determination was based on the previous study (Sieuwerts et al., 2008). The 16S rRNA sequences of the bacteria in FF are shown in Supplementary Figure S1. After fermentation, the fermented corn-soybean meal was dried at 30–40°C to a moisture content of 10%. The ingredients and nutrient content (%) of the UF and FF are listed in Table 1. Both the UF and FF met all recommended nutrient levels (NRC, 2012), and neither contained antimicrobials or growth promoters.

**TABLE 1:** Ingredients and nutrient components of the experimental meals.

**Ingredients**	**Ratio/%**		**Nutrient component^2^**	**Content/%**	
			
	**UF**	**FF**		**UF**	**FF**
Corn	67.0	67.0	Dry matter	89.42	76.03
Soybean meal	21.0	21.0	Gross energy	12.78	12.65
Wheat bran	8.0	8.0	Crude protein,	15.21	16.36
Premix^1^	4.0	4.0	Crude fiber	2.48	2.96
Total	100	100	Crude fat,	2.02	1.40
			Crude ash	5.95	5.21
			Acid Detergent Fiber	4.04	4.02
			Neutral detergent fiber	13.24	11.78

*^1^Premix supplied per kilogram of meal: VD_3_, 2800 IU; VE, 26 mg; VK, 2 mg; VB1, 50 mg; VB6, 3 mg; VA, 6480 IU; VB2, 4 mg; VB12, 0.03 mg; pantothenic acid, 9 mg; nicotinic acid, 20 mg; choline chloride, 300 mg; biotin, 0.2 mg; Fe, 200 mg; Cu, 95 mg; Mn, 30 mg; folic acid, 1.2 mg; Zn 100 mg; I, 0.35 mg; Se, 0.36 mg; P 0.1%; NaCl, 0.5%; lysine, 0.1%; Ca, 0.9%. ^2^Nutrient contents are calculated values.*

### Animals, Housing, and Treatment

A total of 48 growing barrow pigs (Duroc × Landrace × Large White) (53.90 ± 1.31 kg initial body weight) were randomly allocated into one of two feeding groups; one group was fed with commercial soybean meal (UF) and a second with fermented complete commercial soybean meal (FF). Each group consisted of 24 barrow pigs that were housed in six pens, with four pigs per pen, in an environmentally controlled facility under a constant temperature of 25–28°C and with free access to feed and clean water throughout the experimental period.

### Sample Collection and Preparation

Upon reaching slaughter weight (approximately 110 kg), the control group was fed UF meal for 76 days and the treatment group was fed FF meal for 56 days. One pig from each pen was randomly selected and fasted for 12 h (with free access to water) before slaughter and sampling. Blood samples were collected from the external jugular vein into serum separation tubes and centrifuged at 2,500 rpm for 15 min at 4°C, then stored at −80°C until analysis. After blood sampling, all 12 pigs were anesthetized and slaughtered. After recovery of the duodenum and colon, duodenal and proximal colonic tissue samples were collected, washed with 0.9% saline, quickly frozen, and then stored at −80°C until further analysis. Finally, the digesta in the duodenum and colon was immediately collected and frozen at −80°C.

### Serum Biochemical Indices and Immunoglobulin Analysis

In the present study, the serum concentrations of aspartate transaminase (AST), alanine transaminase (ALT), total protein, triglycerides, glucose, albumin, globulin, and low-density lipoprotein cholesterol were measured using a Hitachi-7180 Biochemical Analyzer (Hitachi Medical Corporation, Tokyo, Japan) provided by the Yangling Demonstration Zone Hospital. Serum concentrations of immunoglobulin (Ig) A, M, and G (IgA, IgM, and IgG, respectively) were determined with a commercial Enzyme-linked immunosorbent assay kit (BIM Biosciences, San Francisco, CA, United States). All procedures were performed in accordance with the manufacturers’ instructions. Each sample was tested in triplicate.

### Total RNA Extraction and Real-Time Polymerase Chain Reaction (PCR)

Total RNA was extracted from liquid nitrogen-frozen samples of the duodenum and colon using TRIzol Regent (Takara Bio Inc., Shiga, Japan). RNA integrity and quality were determined by agarose gel electrophoresis (1%) and spectrometry (A260/A280), respectively. A commercial reverse transcription (RT) kit (Takara Bio Inc.) was used for the synthesis of cDNA. The RT products (cDNA) were stored at −20°C for relative quantification by PCR. For a real-time quantitative polymerase chain reaction (RT-qPCR), every reaction was performed in triplicate using SYBR green kits on an Applied Biosystems ABI 7500 system (Thermo Fisher Scientific, Waltham, MA, United States). The expression levels of all genes were normalized to that of glyceraldehyde 3-phosphate dehydrogenase (GAPDH) using the 2^–ΔΔCT^ method. The sequences of primers used for RT-qPCR are listed in Table 2.

**TABLE 2:** Primers sequences for RT-qPCR.

**Genes**	**Primer sequence (5′-3′)**	**Product (bp)**	**GenBank accession**
*TLR1*	F:TTAGGAGACTCTTACGGGGAA	135	NM_001031775.1
	R:ATTTACTGCGGTGCTGACTGA		
*TLR2*	F:GTTTTACGGAAATTGTGAAACTG	128	NM_213761.1
	R:TCCACATTACCGAGGGATTT		
*TLR3*	F:GCATTGCCTGGTTTGTTAGTTG	122	NM_001097444.1
	R:TGTATCAAAAAGAATCACTGGGAG		
*TLR4*	F:ATATGGCAGAGGTGAAAGCAC	125	NM_001113039.2
	R:GAAGGCAGAGATGAAAAGGGG		
*TLR5*	F:AGTTCCGGGGATTTTGTTTCA	110	NM_001348771.1
	R:GCATAAGTAGGCATCGTATTTGTAT		
*TLR6*	F:CATCACCAGCCTCAAGCATTT	90	NM_213760.2
	R:TTCAGTTGTGTCAAGTTGCCAA		
*TLR7*	F:ATAGCGAGCATCACTCCAGCC	127	NM_001097434.1
	R:TAATCTGCTGCCTTCTGGTGC		
*TLR8*	F:CTGGGATGCTTGGTTCATCT	150	NM_214187.1
	R:CATGAGGTTGTCGATGATGG		
*TLR9*	F:ACAATGACATCCATAGCCGAGT	80	NM_213958.1
	R:CAGATCGTTGCCGCTAAAGT		
*PBD-1*	F:TGCCACAGGTGCCGATCT	105	NM_213838.1
	R:CTGTTAGCTGCTTAAGGAATAAAGGC		
*PR39*	F:CCACTCCATCACCGTTTTCC	129	NM_214450.1
	R:CAAGGCCACCTCCGTTTT		
*GAPDH*	F:AGGTCGGAGTGAACGGATTTG	118	NM_001206359.1
	R:ACCATGTAGTGGAGGTCAATGAAG		

*F, forward; R, reverse; PBD-1, porcine beta-defensin 1; PR39, proline-arginine rich 39-amino acid peptide; TLR, Toll-like receptor; GAPDH, glyceraldehyde-3-phosphate dehydrogenase.*

### Intestinal Digesta DNA Extraction and Pyrosequencing

Total genomic DNA was extracted from the duodenal and colonic digesta samples using the cetyltrimethylammonium bromide and sodium dodecyl sulfate method. DNA concentration and purity were monitored on 1% agarose gels. DNA extracted from each sample was used as a template to amplify the V4–V5 regions of 16S rRNA genes for later pyrosequencing by Novogene Biological Information Technology Co. (Beijing, China), as described previously (Tian and Zhang, 2017). The forward and reverse primer sequences for the V4–V5 rRNA gene library preparation are presented in Supplementary Table S1. Raw reads were submitted to the Sequence Read Archive of the National Center for Biotechnology Information (SRA, No. PRJNA524989).

### Bioinformatics Analyses

The paired-end reads were merged using FLASH (V1.2.7)^1^ (Magoc and Salzberg, 2011). Quality filtering on the raw tags was performed according to the QIIME 1.7.0^2^ quality controlled process (Caporaso et al., 2010). Next, the tags were compared with the reference database (Gold database)^3^ (Edgar et al., 2011) using an UCHIME algorithm (UCHIME Algorithm)^4^, to detect chimera sequences, and the chimera sequences were then removed (Haas et al., 2011). The Effective Tags were then finally obtained. High-quality clean tags were obtained and classified into the same operational taxonomic units (OTUs) at an identity threshold of 97% similarity by UPARSE software (Uparse v7.0.1001)^5^ (Edgar, 2013). For each representative sequence, the Greengene Database^6^ (DeSantis et al., 2006) was used based on a ribosomal database projects (RDPs) classifier (Version 2.2)^7^ (Wang et al., 2007) algorithm to annotate taxonomic information. In order to study the phylogenetic relationship of different OTUs, and the difference of the dominant species in different samples (groups), multiple sequence alignments were conducted using MUSCLE software (Version 3.8.31)^8^ (Edgar, 2004). OTUs abundance information was normalized using a standard of sequence number corresponding to the sample with the fewest number of sequences. Subsequent analyses of alpha diversity and beta diversity were all performed based on this normalized output data. Alpha diversity is applied in analyzing the complexity of species diversity for a sample through four indices, including Observed species, Chao 1, Shannon, Simpson, and ACE. All the indices in our samples were calculated with QIIME (Version 1.7.0) and displayed with R software (Version 2.15.3). The relative abundance at the phylum and genus levels was compared between the two groups, the top 30 most abundant families were defined as predominant genera and sorted for the comparison.

### Untargeted Metabolomics Study Based on Liquid Chromatography Tandem Mass Spectrometry (LC-MS/MS)

Tissues (100 mg) were individually ground with liquid nitrogen and the homogenate was resuspended in pre-chilled 80% methanol (−20°C) and then vortexed. The samples were incubated at −20°C for 60 min and then centrifuged at 14,000 *g* and 4°C for 20 min. The supernatants were subsequently transferred to a fresh Eppendorf tube and spun in a vacuum concentrator until dry. The dried metabolite pellets were reconstituted in 60% methanol and analyzed by LC-MS/MS.

LC-MS/MS analyses were performed using a Vanquish ultra-high-performance liquid chromatography (UHPLC) system (Thermo Fisher Scientific) coupled with an Orbitrap Q Exactive HF-X mass spectrometer (Thermo Fisher Scientific) at Novogene Genetics, Beijing, China. Samples were injected into a Hypersil Gold column (100 × 2.1 mm, 1.9 μm; Thermo Fisher Scientific) using a 16 min linear gradient at a flow rate of 0.3 mL/min. The eluents for the positive polarity mode were eluent A (0.1% aqueous formic acid solution) and eluent B (methanol). The eluents for the negative polarity mode were eluent A (5 mmol/L ammonium acetate, pH 9.0) and eluent B (methanol). The solvent gradient was set as follows: 2% B, 1.5 min; 2–100% B, 12.0 min; 100% B, 14.0 min; 100–2% B, 14.1 min; and 2% B, 16.0 min. A Q Exactive HF-X mass spectrometer (Thermo Fisher Scientific) was operated in positive/negative polarity mode with a spray voltage of 3.2 kV, capillary temperature of 320°C, sheath gas flow rate of 35 arb, and auxiliary gas flow rate of 10 arb.

The raw data files generated by UHPLC-MS/MS were processed using the Compound Discoverer 3.0 (Thermo Fisher Scientific) to perform peak alignment, peak picking, and quantitation of each metabolite. The main parameters were set as follows: retention time tolerance, 0.2 min; actual mass tolerance, 5 ppm; signal intensity tolerance, 30%; signal/noise ratio, 3; and minimum intensity, 100000. Afterward, peak intensities were normalized to the total spectral intensity. The normalized data were used to predict the molecular formula based on additive ions, molecular ion peaks, and fragment ions. Then, the peaks were matched with the mzCloud^9^ and ChemSpider^10^ databases to obtain accurate qualitative and relative quantitative results. The online KEGG database was used to identify metabolites by matching the molecular mass data. Finally, metabolites for separating the models were selected with the following requirements: variable importance in projection (VIP) > 1 and | P(corr)| ≥ 0.5 with 95% jack-knifed confidence intervals. The Student’s *t*-test was applied to further analyze intergroup significance of the selected metabolites. Pathway analysis and enrichment analysis of differential metabolites were conducted on the MetaboAnalyst web server^11^.

### Statistical Analysis

Data are presented as the mean ± standard error of the mean (SEM). All statistical analyses were performed using IBM SPSS Statistics for Windows, version 19.0 (IBM Corporation, Armonk, NY, United States) using an one-way ANOVA with Turkey’s multiple comparison test or Student’s test. A probability (*P*) value of <0.05 was considered statistically significant. The correlations between the colonic microbial composition (relative abundance of family higher than 0.1%) and serum immunity and genes related to gut immunity that were significantly affected by FF treatment were assessed by a Spearman’s correlation test using GraphPad Prism version 8.00 (GraphPad Software, San Diego, CA, United States). The correlations between the colonic microbial composition (relative abundance of family higher than 0.1%) and metabolites that were significantly affected by FF meal were assessed by a Spearman’s correlation analysis using GraphPad Prism version 8.00 (GraphPad Software, San Diego, CA, United States).

## Results

### FF Increased Growth Performance

To investigate the effect of FF on body weight, after reaching the similar weight (53.19 ± 2.17 kg vs. 54.60 ± 1.62 kg), the pigs fed a corn-soybean meal were given free access to FF or UF. When compared with the UF group, the average daily weight gain was significantly increased in the FF group (*P* < 0.05) and there was no significant difference (*P* > 0.05) in the average daily intake among the two groups. Furthermore, the weight gain to food ratio was significantly increased in the FF group rather than in the UF group (*P* < 0.05) (Table 3).

**TABLE 3:** Effects of fermented meal on growth performance of grower-finisher pigs.

**Items**	**UF**	**FF**	***P*-value**
Initial body weight (kg)	53.19 ± 2.17	54.60 ± 1.62	0.615
Final body weight (kg)	115.34 ± 2.33	116.76 ± 2.04	0.606
Average daily gain (kg⋅d^–1^)	0.74 ± 0.06	1.04 ± 0.04	0.013
Average daily intake (kg⋅d^–1^)	2.20 ± 0.07	2.31 ± 0.07	0.350
Gain:food ratio	0.33 ± 0.02	0.45 ± 0.03	0.021

*Data are presented as the mean ± SEM (*n* = 6). UF, pigs fed with unfermented corn-soybean meal; FF, pigs fed with fermented corn-soybean meal.*

### FF Changed Serum Immunity

**FIGURE 1:**
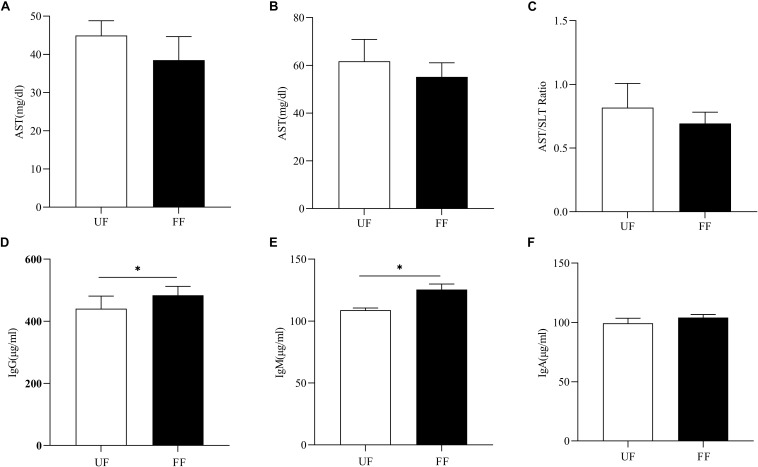
FF changed serum immunity. **(A)** AST, **(B)** ALT, **(C)** AST/ALT ratio, **(D–F)**. Serum immunoglobulins, IgM **(D)**, IgA **(E)**, and IgG **(F)**. UF, pigs fed with unfermented corn-soybean meal; FF, pigs fed with fermented corn-soybean meal. ^∗^*P* < 0.05.

Next, the effects of FF on immunity were assessed. As shown in Figures 1A–C, the ALT and AST concentrations tended to decrease in the FF-treated group as compared to the UF group (*P* > 0.05). There were no significant differences in the other serum parameters between the two groups (Supplementary Table S2). As shown in Figures 1D–F, FF significantly increased the serum concentrations of IgM and IgG (*P* < 0.05), while there was no significant difference in IgA levels between the two meals.

### Effect of FF on the Expression of Genes Related to Gut Immunity

**FIGURE 2:**
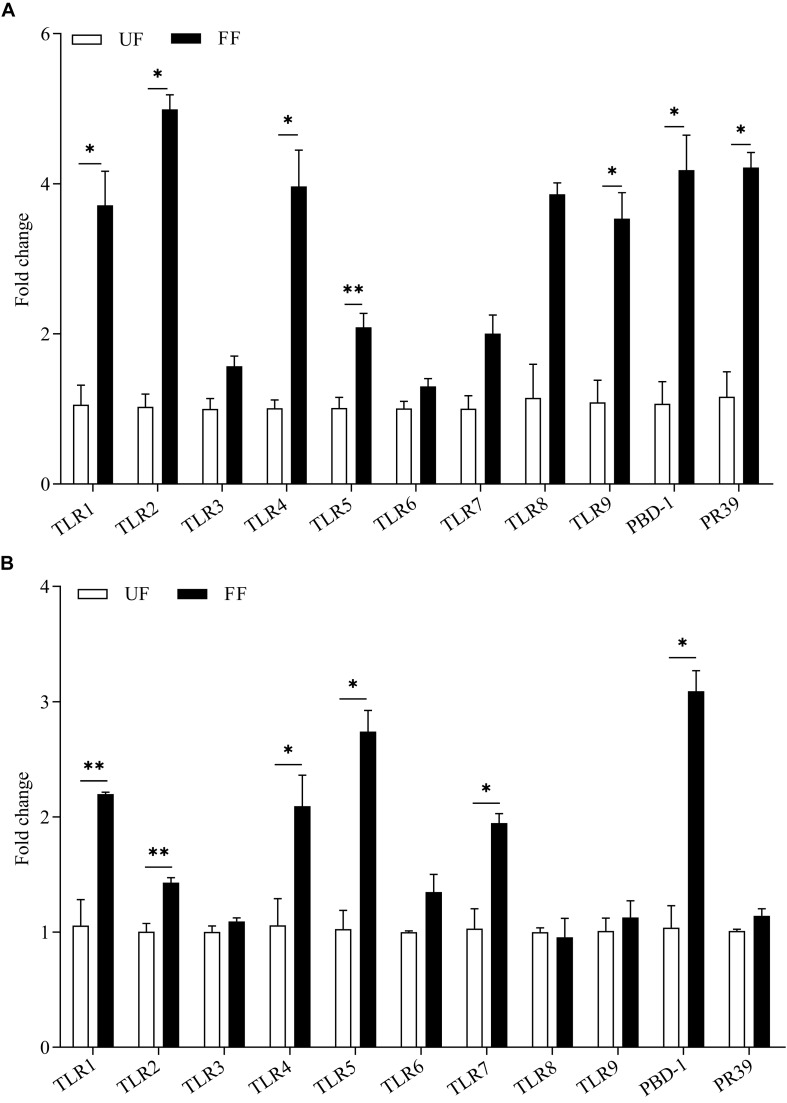
Expression levels of TLR genes and antimicrobial peptides in the duodenum **(A)** and colon **(B)**. The UF group was designed as one-fold change. UF, pigs fed with unfermented corn-soybean meal; FF, pigs fed with fermented corn-soybean meal. Data are presented as the mean ± SEM (*n* = 6). ^∗^*P* < 0.05 and ^∗∗^*P* < 0.01.

The effects of FF on the expression of genes related to gut immunity was determined. The expression levels of the antimicrobial peptide-encoding genes *PBD-1* and *PR39* revealed the potential capacity for the eradication of invading pathogens. The results of the gene expression analysis of the intestinal tissue are presented in Figure 2. The FF meal significantly increased the mRNA abundance of AMPs and TLRs. In the duodenum, the mRNA abundances of *TLR1*, *TLR2*, *TLR3*, *TLR4*, *PBD-1*, and *PR-39* in the FF-treated pigs were significantly increased (*P* < 0.05), while those of *TLR5* and *TLR9* were very significantly increased (*P* < 0.01; Figure 2A). In the proximal colon, the mRNA abundances of *TLR4* and *TLR5* in the FF-treated pigs were significantly increased (*P* < 0.05), while those of *TLR1*, *TLR2*, *TLR7*, and *PBD-1* in the FF-treated pigs were very significantly increased (*P* < 0.01; Figure 2B).

### FF Shaped the Intestinal Microbiota

To explore the cause of improved immunity in FF-treated pigs, the intestinal microbiota were quantified by 16S rRNA sequence analysis, which identified a total of 65,249 ± 16,236 V4–V5 16S rRNA sequence reads from each sample. Binning sequences using a pairwise identity threshold of 97% obtained an average of 800 ± 231 operational taxonomic units per sample. MOTHUR plotting for the construction of a sparse curve between the number of reads and the number of operational taxonomic units revealed a tendency for plateau saturation (Supplementary Figure S2). Statistical estimates of species richness for 5,000 subsets of each sample at a genetic distance of 3% showed that there was no difference in richness estimators (ACE and Chao 1) and the diversity indices (Shannon and Simpson diversity) (Table 4).

**TABLE 4:** Diversity estimation of the 16S rRNA gene libraries from microbiota in the duodenum and colon of pigs fed the control UF and FF meals.

**Items**	**Duodenum**			**Colon**		
		
	**UF**	**FF**	***P*-value**	**UF**	**FF**	***P*-value**
Ace	734.452 ± 255.71	563.738 ± 156.54	0.142	1116.32 ± 84.48	968.950 ± 45.31	0.132
Chao 1	719.902 ± 237.37	542.699 ± 149.85	0.148	1120.88 ± 83.80	970.55 ± 42.16	0.201
Shannon	3.573 ± 0.86	3.132 ± 1.79	0.191	7.63 ± 0.31	7.12 ± 0.86	0.918
Simpson	0.721 ± 0.08	0.637 ± 0.31	0.332	0.99 ± 0.04	0.96 ± 0.07	0.993

*UF, pigs fed with unfermented corn-soybean meal; FF, pigs fed with fermented corn-soybean meal. Data are presented as the mean ± SEM (*n* = 6).*

At the phylum level, Firmicutes and Bacteroidetes were the most predominant phyla in the duodenum and colon (Tables 5, 6). FF meal significantly decreased the proportion of Bacteroidetes and Verrucomicrobia (*P* < 0.05) and tended to decrease the relative abundance of Spirochaetes in the duodenum (*P* = 0.069) (Table 5). FF meal significantly increased the proportion of Firmicutes and Actinobacteria (*P* < 0.01), whereas the proportion of Proteobacteria, Bacteroidetes, and Verrucomicrobia was decreased in the colon (*P* < 0.05) (Table 6).

**TABLE 5:** Relative abundance of microbial phylum (percentage) in the duodenum of pigs in the FF and control groups.

**Items**	**UF**	**FF**	***P*-value**
Firmicutes	51.29 ± 11.11	78.61 ± 14.67	0.168
Proteobacteria	1.15 ± 0.54	17.28 ± 15.35	0.341
Bacteroidetes	33.26 ± 11.16	1.10 ± 0.37	0.035
Actinobacteria	0.95 ± 0.81	2.06 ± 0.84	0.363
Unclassified phylum	11.18 ± 7.16	0.02 ± 0.01	0.180
Tenericutes	1.22 ± 0.63	0.47 ± 0.04	0.348
Planctomycetes	0.01 ± 0.01	0.05 ± 0.03	0.366
Verrucomicrobia	0.07 ± 0.02	0.01 ± 0.00	0.037
Euryarchaeota	0.01 ± 0.00	0.01 ± 0.00	0.397
Spirochaetes	0.64 ± 0.26	0.03 ± 0.01	0.069

*UF, pigs fed with unfermented corn-soybean meal; FF, pigs fed with fermented corn-soybean meal. Data are presented as the mean ± SEM (*n* = 6).*

**TABLE 6:** Relative abundance of microbial phylum (percentage) in the colon of pigs in the FF and control groups.

**Items**	**UF**	**FF**	***P*-value**
Firmicutes	40.42 ± 1.94	62.64 ± 3.52	0.001
Proteobacteria	1.68 ± 0.13	0.20 ± 0.05	<0.001
Bacteroidetes	51.59 ± 2.29	31.78 ± 3.27	0.001
Actinobacteria	0.15 ± 0.02	0.50 ± 0.10	0.020
Unclassified phylum	0.08 ± 0.02	0.04 ± 0.01	0.085
Tenericutes	2.27 ± 0.33	1.59 ± 0.23	0.129
Planctomycetes	0.02 ± 0.01	0.49 ± 0.23	0.095
Verrucomicrobia	0.57 ± 0.12	0.01 ± 0.00	0.005
Euryarchaeota	0.11 ± 0.03	0.18 ± 0.15	0.689
Spirochaetes	2.59 ± 0.30	2.50 ± 0.63	0.910

*UF, pigs fed with normal commercial feed; FF, pigs fed with fermented meal. Data are presented as the mean ± SEM (*n* = 6).*

A family-level analysis of the top 30 most abundant families revealed that Acidaminococcaceae and Clostridiales_vadinBB60_group were significantly decreased in relative abundance by the FF meal treatment in the duodenum (*P* < 0.05) (Table 7). A family-level analysis of the top 30 most abundant families revealed that Ruminococcaceae, Rikenellaceae, Christensenellaceae, Lactobacillaceae, and Family XIII were significantly increased in relative abundance by the FF meal treatment, whereas the abundance of Prevotellaceae, Lachnospiraceae, Clostridiaceae_1, Bacteroidales_RF16_group, Streptococcaceae, Veillonellaceae, Erysipelotrichaceae, Peptostreptococcaceae, Acidaminococcaceae, Bacteroidaceae, Succinivibrionaceae, Clostridiales_vadinBB60_group, Anaeroplasmataceae, Alcaligenaceae, Fibrobacteraceae, Rhodospirillaceae, and Unidentified_Thermoplasmatales was decreased in the colon (*P* < 0.05) (Table 8).

**TABLE 7:** Relative abundance (percentage) for the top 30 most abundant family in the duodenum of pigs in the fermented feed (FF) and control (UF) groups.

**Items**	**UF**	**FF**	***P*-value**
Prevotellaceae	23.85 ± 10.94	0.33 ± 0.13	0.084
Clostridiaceae_1	13.72 ± 9.68	7.26 ± 5.81	0.582
Unclassified family	12.74 ± 6.79	0.79 ± 0.46	0.139
Lactobacillaceae	11.52 ± 10.01	42.17 ± 15.73	0.137
Ruminococcaceae	10.63 ± 6.04	4.75 ± 4.22	0.446
Veillonellaceae	5.54 ± 2.40	8.78 ± 3.60	0.474
Lachnospiraceae	4.55 ± 1.50	1.84 ± 1.61	0.246
Bacteroidales_S24-7_group	3.87 ± 2.15	0.22 ± 0.14	0.150
Rikenellaceae	2.16 ± 0.97	0.10 ± 0.03	0.087
Porphyromonadaceae	1.34 ± 0.66	0.11 ± 0.04	0.119
Peptostreptococcaceae	1.28 ± 0.38	4.57 ± 3.28	0.362
Bacteroidales_RF16_group	1.21 ± 0.64	0.04 ± 0.02	0.125
Erysipelotrichaceae	0.99 ± 0.27	3.67 ± 2.48	0.329
Nocardiaceae	0.78 ± 0.78	0.03 ± 0.02	0.381
Streptococcaceae	0.74 ± 0.30	0.18 ± 0.08	0.122
Acidaminococcaceae	0.74 ± 0.16	0.06 ± 0.02	0.009
Spirochaetaceae	0.64 ± 0.26	0.03 ± 0.01	0.069
Christensenellaceae	0.64 ± 0.33	1.08 ± 0.96	0.681
Family XIII	0.52 ± 0.34	0.40 ± 0.36	0.823
Bacteroidaceae	0.40 ± 0.21	0.04 ± 0.01	0.147
Campylobacteraceae	0.20 ± 0.10	0.01 ± 0.01	0.131
Mycoplasmataceae	0.19 ± 0.17	0.00 ± 0.00	0.296
Neisseriaceae	0.18 ± 0.12	0.07 ± 0.04	0.414
Alcaligenaceae	0.17 ± 0.11	0.02 ± 0.01	0.240
Clostridiales_vadinBB60_group	0.13 ± 0.05	0.00 ± 0.00	0.040
Burkholderiaceae	0.12 ± 0.12	0.01 ± 0.00	0.370
Anaeroplasmataceae	0.11 ± 0.05	0.00 ± 0.00	0.063
Peptococcaceae	0.11 ± 0.07	0.02 ± 0.02	0.269
Helicobacteraceae	0.10 ± 0.05	0.00 ± 0.00	0.147
P-2534-18B5_gut_group	0.09 ± 0.03	0.01 ± 0.00	0.060

*UF, pigs fed with unfermented corn-soybean meal; FF, pigs fed with fermented corn-soybean meal. Data are presented as the mean ± SEM (*n* = 6).*

**TABLE 8:** Relative abundance (percentage) for the top 30 most abundant family in the colon of pigs in the fermented feed (FF) and control (UF) groups.

**Items**	**UF**	**FF**	***P*-value**
Prevotellaceae	28.31 ± 4.11	9.54 ± 1.13	0.005
Ruminococcaceae	13.22 ± 1.18	31.78 ± 3.48	0.002
Lachnospiraceae	11.77 ± 0.82	8.41 ± 0.84	0.017
Bacteroidales_S24-7_group	11.29 ± 1.47	8.18 ± 0.73	0.099
Rikenellaceae	4.63 ± 0.25	8.89 ± 1.27	0.020
Unclassified family	3.97 ± 0.42	2.52 ± 0.31	0.022
Clostridiaceae_1	3.43 ± 0.49	0.69 ± 0.14	0.002
Bacteroidales_RF16_group	3.12 ± 0.36	1.50 ± 0.29	0.006
Streptococcaceae	2.92 ± 0.92	0.01 ± 0.01	0.025
Spirochaetaceae	2.59 ± 0.30	2.50 ± 0.63	0.910
Veillonellaceae	2.42 ± 0.39	0.16 ± 0.05	0.002
Porphyromonadaceae	2.09 ± 0.30	1.84 ± 0.40	0.628
Erysipelotrichaceae	1.45 ± 0.07	0.87 ± 0.12	0.002
Peptostreptococcaceae	1.32 ± 0.13	0.91 ± 0.12	0.040
Acidaminococcaceae	1.20 ± 0.11	0.57 ± 0.12	0.004
Bacteroidaceae	0.95 ± 0.15	0.38 ± 1.44	0.012
Christensenellaceae	0.86 ± 0.17	7.36 ± 6.65	0.006
Lactobacillaceae	0.66 ± 0.17	9.95 ± 1.44	0.221
Family XIII	0.45 ± 0.04	1.16 ± 0.16	0.006
Succinivibrionaceae	0.40 ± 0.10	0.00 ± 0.00	0.010
Clostridiales_vadinBB60_group	0.40 ± 0.04	0.04 ± 0.01	<0.001
Anaeroplasmataceae	0.39 ± 0.04	0.02 ± 0.01	<0.001
Bacteroidales_BS11_gut_group	0.30 ± 0.12	0.58 ± 0.33	0.450
Alcaligenaceae	0.27 ± 0.03	0.01 ± 0.00	<0.001
Campylobacteraceae	0.21 ± 0.09	0.01 ± 0.00	0.072
P-2534-18B5_gut_group	0.20 ± 0.04	0.31 ± 0.03	0.065
Fibrobacteraceae	0.14 ± 0.03	0.03 ± 0.01	0.019
Rhodospirillaceae	0.14 ± 0.03	0.01 ± 0.00	0.003
Peptococcaceae	0.11 ± 0.02	0.15 ± 0.02	0.186
Unidentified_Thermoplasmatales	0.10 ± 0.03	0.00 ± 0.00	0.017

*UF, pigs fed with normal commercial feed; FF, pigs fed with fermented meal. Data are presented as the mean ± SEM (*n* = 6).*

### Correlation Between Colonic Microbial Composition and Serum Immunity and Genes Related to Gut Immunity

**FIGURE 3:**
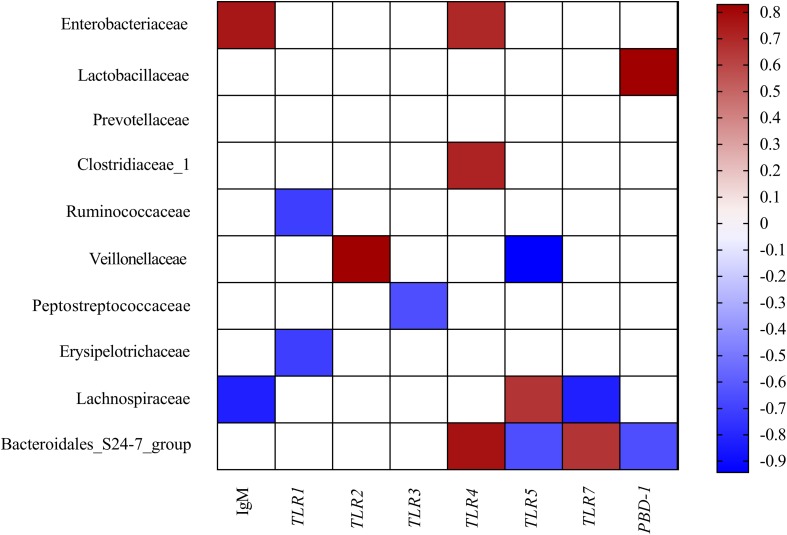
Correlation analysis between colonic microbial composition (relative abundance of family higher than 0.1%) and serum immunity and genes related to gut immunity that were significantly affected by FF treatment in the colon of pigs. The color represents a significant correlation (*P* < 0.05), and the intensity of the colors represents the degree of association. Red represents significant positive correlation and blue represents significantly negative correlation (*n* = 6).

To comprehensively analyze the relations between host serum immunity, genes related to gut immunity, and gut microbiota, a correlation matrix was generated by calculating the Spearman’s correlation coefficient. As shown in Figure 3, Spearman’s correlation analysis showed that the serum IgM was positively associated with the abundance of Enterobacteriaceae but negatively related to the proportion of Lachnospiraceae in the colon. The gene expression of *TLR1* was negatively correlated with the abundance of Ruminococcaceae and Erysipelotrichaceae. The gene expression of *TLR2* was positively associated with the abundance of Veillonellaceae. The gene expression of *TLR3* was negatively correlated with the abundance of Peptostreptococcaceae. The gene expression of *TLR4* was positively correlated with the abundance of Enterobacteriaceae, Clostridiaceae_1, and Bacteroidales_S24-7_group. The gene expression of *TLR5* and *PBD-1* were positively associated with the abundance Lachnospiraceae but negatively related to the proportion of Bacteroidales_S24-7_group. The gene expression of *TLR7* was positively associated with the abundance Bacteroidales_S24-7_group but negatively related to the proportion of Lachnospiraceae. Additionally, the gene expression of *TLR5* was also negatively related to the proportion of Veilonellaceae.

### Effect of FF on Fermentation Metabolites in Colonic Digesta

**FIGURE 4:**
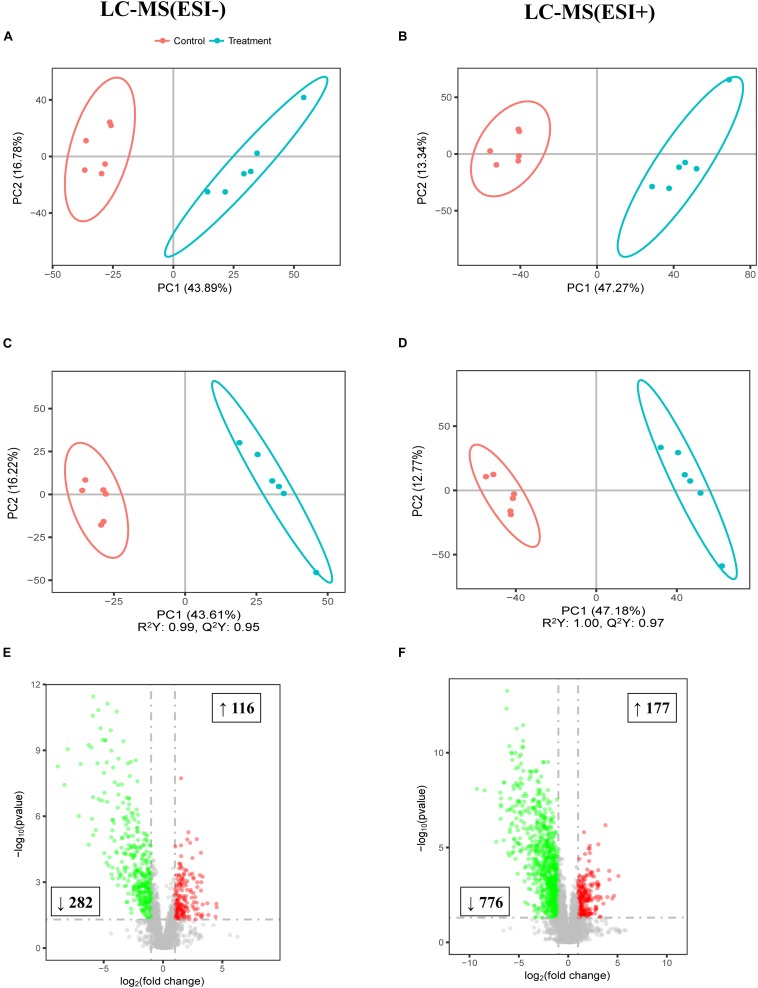
Multivariate statistical analysis of untargeted metabolomics data obtained using the LC-MS/MS approach. PCA score plot of colonic metabolomics data for treatment (blue) and control (red) pigs obtained by **(A)** LC-MS (ESI−) and **(B)** LC-MS (ESI+) (*n* = 6). **(C)** PLS-DA score plot of colonic metabolomics data obtained by LC-MS (ESI−); R^2^Y = 0.99; Q^2^ = 0.95. **(D)** PLS-DA score plot of colonic metabolomics data obtained by LC-MS (ESI+) data; R^2^Y = 1.00; Q^2^ = 0.97. **(E)** Score plot of LC-MS (ESI−) data with 2,395 metabolite signals detected. **(F)** Score plot of LC-MS (ESI+) data with 5,708 metabolite signals detected. Red circles in volcano plots are model-separated metabolites following the conditions of VIP > 1 and | P(corr)| ≥ 0.5 with 95% jack-knifed confidence intervals. Red or green rectangles indicate the numbers and tendency of metabolites to separate in the model when FF group pigs are compared with UF group.

To further study the effect of FF on the gut, the colonic digesta metabolic profiles of the two meals were acquired by LC-MS. As shown by the PCA score plots presented in Figures 4A,B which distinguished metabolic communities based on meal of colonic sampling, the metabolic communities were clustered. The partial least squares discriminant analysis (PLS-DA) score plots also reflected that FF led to significant biochemical changes (Figures 4C,D). Furthermore, LC-MS/MS (ESI−) and LC-MS/MS (ESI+) detected a total of 398 and 953 biomarker metabolites, respectively (Figures 4E,F).

**FIGURE 5:**
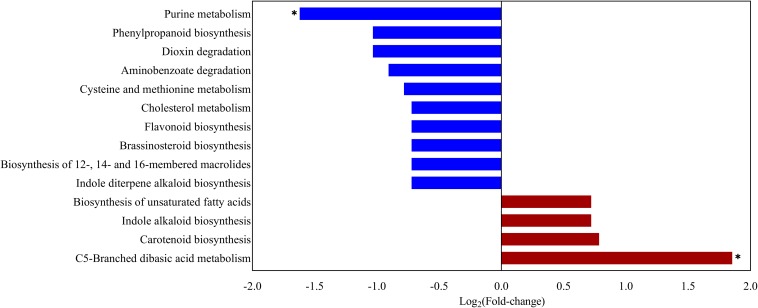
KEGG pathway enrichment of the changed metabolites. Overview of metabolites that were enriched in pigs fed the FF meal compared to the UF meal. Length intensity indicates the log_2_-fold change in magnitude, using red for upregulation and blue for downregulation (^∗^*P* < 0.05).

In summary, there were significant differences in the 1,351 biomarker metabolites between the UF and FF groups (detailed information is presented in Supplementary Data S1). Next, the Kyoto Encyclopedia of Genes and Genomes (KEGG) was used to analyze the pathways of the metabolites that differed between the two meal groups (Figure 5). The effects of C5-branched dibasic acid metabolism were significantly upregulated while the purine metabolism was significantly downregulated in the FF group, as compared to the control group (*P* < 0.05). Meanwhile, the effects of dioxin degradation, phenylpropanoid biosynthesis, and aminobenzoate degradation were downregulated in the FF group (0.05 < *P* < 0.1).

### Correlation Between Microbiota Communities and Related Metabolites

**FIGURE 6:**
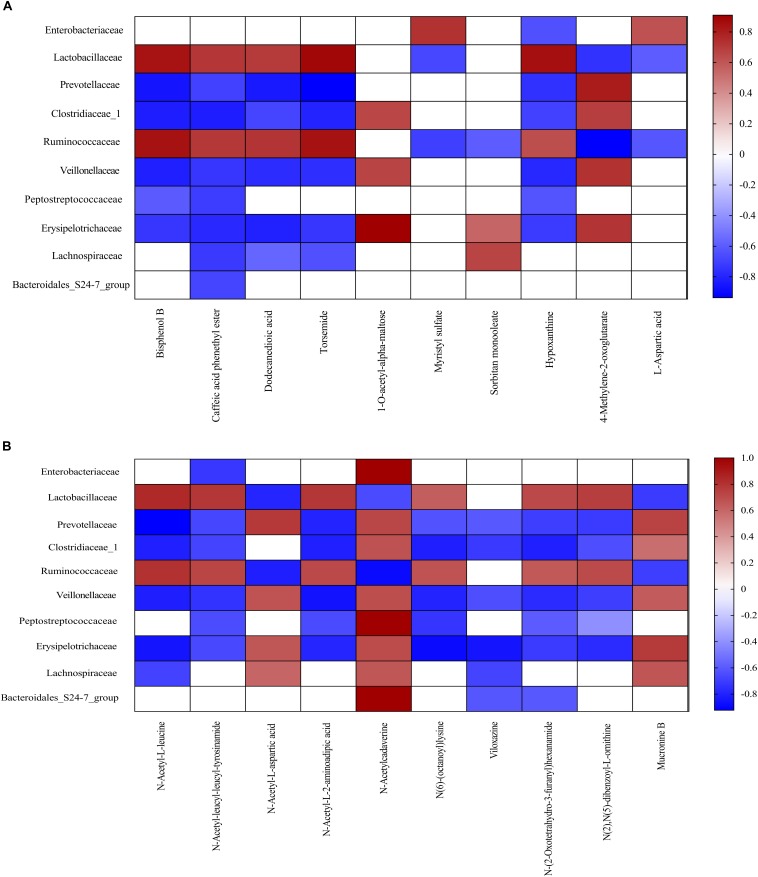
Correlation analysis between colonic metabolites and colonic bacterial family-level taxa in pigs fed the FF meal compared to the UF meal. **(A)** LC-MS (ESI–). **(B)** LC-MS (ESI+). The color represents a significant correlation (*P* < 0.05), and the intensity of the colors represents the degree of association. Red represents significant positive correlation and blue represents significantly negative correlation (*n* = 6).

Metabolomics has been shown to be an important tool to reveal the potential crosstalk of host and gut microbiota. Therefore, correlations between metabolites and family-level microbiota with significant differences between two meals were obtained via spearman’s correlation analysis (Figure 6). As shown in Figure 6, ten bacterial strains (family Enterobacteriaceae, Lactobacillaceae, Prevotellaceae, Clostridiaceae_1, Ruminococcaceae, Veillonellaceae, Peptostreptococcaceae, Erysipelotrichaceae, Lachnospiraceae, and Bacteroidales_S24-7_group) were most closely related to the metabolites in the FF group, as compared to the control group (*P* < 0.05).

## Discussion

The gut microbiota are critical to metabolism, nutrient absorption, and host immunity (El Aidy et al., 2013), and the pig microbiota has become the focus of much attention (Frese et al., 2015; Ji et al., 2018; Wang W. et al., 2019). FF, as an available feed alternative, has great potential to improve gut health and maintain gastrointestinal tract microbial homeostasis (Jin et al., 2017) and could modulate the host gut microbiota through dietary manipulation (Wang et al., 2018). In this study, the effects of FF versus normal feed on serum immunity, expression of genes related to gut immunity, gut microbiota composition, and bacterial metabolites were investigated in grower-finisher pigs. The results reflected that FF regulated the microbiota composition in the duodenum and colon of pigs, and it also selectively changed the metabolomics profiles.

Corn-soybean meal is the most frequently used meal for livestock production in China. Solid-state fermentation can improve the nutritional value of plant materials and has it been suggested to increase the use of FF in livestock feeds (Shi et al., 2017). In the animal production, pigs are often slaughtered at a constant body weight (market weight, around 110 kg) to maintain uniformity of pork products and maximize profits (Kim et al., 2005; Frederick et al., 2006; Vermeer et al., 2014). In the present study, we performed the study at an average initial body weight (53.19 ± 2.17 vs. 54.60 ± 1.62 kg), and an interesting finding in this study was that FF meal greatly reduced the time to market (76 vs. 56 days). As compared with the UF group, the average daily weight gain and weight gain: food ratio in the FF group were significant increased (*P* < 0.05), which reflects improved growth performance and feed conversion efficiency, in accordance with the findings of previous studies (Canibe and Jensen, 2003). The fermentation process is believed to promote functional activities, such as antimicrobial and antioxidant activities, and increases the production of growth factors, hormones, and amino acids (Ng et al., 2011; Laskowska et al., 2017).

Proper function of the immune system is important for grower-finisher pigs. Immunoglobulins are immune-active molecules that play important roles in the humoral immune response (Kong et al., 2007). In the present study, the concentrations of IgG and IgM were significantly greater in the FF group (*P* < 0.05). The levels of IgG reflected immune status (Machado-Neto et al., 1987). IgM is associated with anti-inflammation and a higher concentration reflects better immune status (Vaschetto et al., 2017). Our results were similar to those of Zhu et al. (2017), who reported that weaned piglets fed fermented soybean meal had higher serum concentrations of IgG and IgM, as compared with the controls. Some probiotic strains can be used as immunomodulators to enhance the concentrations of serum immunoglobulins (Vitini et al., 2000). Laskowska et al. (2017) found that dietary supplementation of Effective Microorganisms activated and enhanced the humoral and cell-mediated immune responses and protected against infection. Although high concentrations of immunoglobulins were observed, there was no intestinal inflammation, suggesting that the immunoglobulins were in the normal ranges. Moreover, FF meal decreased the concentrations of serum ALT and AST (*P* > 0.5). Reportedly, ALT is a liver-specific enzyme and ALT concentrations increased in response to acute liver injury (Robertson et al., 2016). Here, the lower levels of ALT and AST indicated that FF boosted overall health. In short, FF meal enhanced immune performance.

Toll-like receptors are the earliest discovered pattern recognition receptors and play critical roles in innate immunity (Newburg and Walker, 2007; Werling and Coffey, 2007), and TLR expression levels may indicate disease resistance in pigs (Uenishi and Shinkai, 2009; Cheng et al., 2015). Our data showed that the mRNA abundances of TLRs in the FF group were significantly greater than in the control group (*P* < 0.05). In this study, in the duodenum of FF-fed pigs, the mRNA abundances of *PBD-1* and *PR39* were significantly higher than that of the UF control pigs (*P* < 0.05). Meanwhile, the gene expression of *PBD-1* in the colon was also higher than that of the UF meal pigs (*P* < 0.01). These results indicate that FF may benefits the pig gut immunity and in order to fully understand the regulating mechanism of FF on gut immunity, *in vivo* pathogenic challenge model is needed in further studies.

We then used a high-throughput sequencing method based on the 16S rRNA genes to demonstrate the effects of FF on the intestinal microbiota of grower-finisher pigs. In this study, FF had no effect on gut microbial community evenness (ace, Shannon H) and richness (Simpson and Chao 1). Previous studies have shown that probiotic-supplemented FF decreased microbial diversity, which may be linked with improved resistance to gastrointestinal disorders (Ott et al., 2004; He et al., 2017). Similar to previous studies (Kim et al., 2012; Looft et al., 2012; Niu et al., 2015; Li et al., 2017), Firmicutes and Bacteroidetes were the most dominant phyla in the present study. Studies have shown that in obese animals, the ratio of Firmicutes to Bacteroides is increased (Ley et al., 2005, 2006b). In the present study, the ratio of the Firmicutes to Bacteroides was increased in both the duodenum and colon of the treatment group, which indicated that the use of FF changed the proportion of the microbiota and is beneficial to the weight gain of finisher pigs. In addition, a microbiome enriched in Firmicutes has been associated with an increased capacity for energy harvest and obesity (Ley et al., 2006a; Turnbaugh et al., 2006), and an increase of this phylum could therefore increase the amount of calories extracted from the diet. Compared with the control group, the relative abundance of Verrucomicrobia was significantly decreased in the FF group in the duodenum. Verrucomicrobia usually represents a minor population of intestinal microbiota in response to dietary shifts in mice (Pantoja-Feliciano et al., 2013). A relatively smaller proportion of *Proteobacteria* in the FF group was detected in our current study. It’s reported that the members of the phylum Proteobacteria have a low abundance in the gut of healthy humans (Shin et al., 2015) and the increased levels of Proteobacteria may be indicative of a diseased state that commonly occurs during enteric infection or following perturbation of the microbiota (Singh et al., 2015). Moreover, FF significantly changed the gut microbiota composition, as indicated by decreased proportions, and significantly increased the proportions of Actinobacteria (*P* < 0.05). Meanwhile, we found there were five bacterial strains were significantly increased and 17 bacterial strains were significantly decreased in the colon in response to FF meal at the family level (*P* < 0.05); further studies are needed to investigate the roles of these gut bacteria in regulating the swine gut development.

The gut microbiota is important to host health and physiology status (Lalles, 2016). It is reported that microbes in the large intestine undertake more metabolism tasks (Zhao et al., 2015). Using a KEGG pathway analysis, we found that C5-Branched dibasic acid metabolism was significantly upregulated whereas the purine metabolism was significantly downregulated (*P* < 0.05). Similarly, Gomez et al. (2017) measured the fecal microbiota and calculated the functional potential of the microbial communities in healthy and diarrheic calves. They found that C5-branched dibasic acid metabolism was enriched in healthy calves, suggesting that C5-branched dibasic acid metabolism is related to energy generation (Turnbaugh et al., 2006). The purine content of animal feed is a concern because excess intake of purines may increase the risk of hyperuricemia and gout (Zheng et al., 2018). It is necessary to conduct further research beyond the scope of the present study.

Fermented feed meal altered the metabolic functions and phenotypes of gut microbiota in pigs. The relative abundances of bacteria at the family levels were closely associated with the concentration of specific microbial metabolites in the colonic digesta. For instance, Lachnospiraceae is a bacterial family known to be abundant in the intestinal ecosystem (Sagheddu et al., 2016), and it is reported that Lachnospiraceae was positively correlated with carbohydrate metabolism (Morgan et al., 2012). Moreover, we found that Lachnospiraceae was also positively correlated with Sorbitan monooleate, N-Acetyl-L-aspartic acid, N-Acetylcadaverine, and Mucronine B, whereas it negatively correlated with Caffeic acid phenethyl ester, Dodecanedioic acid, Torsemide, N-Acetyl-L-leucine, and Viloxazine. The mechanism underlying the dietary regulation of host phenotypes should be explored with a particular focus on metabolism and related receptors.

Further studies are needed to explore the benefits of FF benefits on the gut microbiota and health of pigs. To this end, fecal microbiota transplantation (FMT) may be an effective strategy. FMT refers to the process of transplantation of fecal bacteria from healthy individuals into a recipient (Drekonja et al., 2015). Growing evidence has shown that the host phenotypes, such as obesity (Lee et al., 2019), Clostridium difficile infection (Khoruts and Sadowsky, 2016), and anti-seizure (Olson et al., 2018) and anti-tumor immunity (Sivan et al., 2015), can be altered by FMT in mammals, demonstrating the critical roles of gut microbiota in host health. Thus, FMT also helped evaluate the potential links between intestinal microbiota and host phenotypes. Recently, Hu et al. (2018) proposed the standardized preparation for FMT for use in pig production and to identify host microbiota-derived bacteriocin targets to determine diarrhea resistance in early-weaned piglets (Hu et al., 2018). In the current study, we determined the FF could benefits the pig’s gut microbiota; it would be interesting to explore the mechanism and we would like to focus on this in our next study.

## Conclusion

In conclusion, we found that long-term consumption of FF meal increased the serum concentrations of IgG and IgM and changed the expression of genes related to gut immunity, which may be associated with alterations in the microbiota community and microbial metabolites and benefits to the pig’s health. In addition, the results suggest that FF altered the microbial composition and modulated the metabolic pathway of microbial metabolism in pig colons. These alterations provide an alternative strategy for improving the intestinal health of pigs.

## Data Availability Statement

The datasets generated for this study can be found in NCBI, No. PRJNA524989.

## Ethics Statement

The use of animals and the performance of all experimental protocols were approved by the Northwest A&F University Animal Welfare Committee (Yangling, Shaanxi Province, China). The processing of animal experiments and sample collection strictly followed the relevant guidelines.

## Author Contributions

XS and JL designed the study, and wrote and revised the manuscript. JL, XZ, HC, QH, BX, and LF helped took samples and performed the experiments and analyses. JL, YL, XL, JH, GY, and XS edited the manuscript. All authors reviewed the final manuscript.

## Conflict of Interest

The authors declare that the research was conducted in the absence of any commercial or financial relationships that could be construed as a potential conflict of interest.

## Footnotes

1 http://ccb.jhu.edu/software/FLASH/

2 http://qiime.org/index.html

3 http://drive5.com/uchime/uchime_download.html

4 http://www.drive5.com/usearch/manual/uchime_algo.html

5 http://drive5.com/uparse/

6 http://greengenes.lbl.gov/Download/

7 http://sourceforge.net/projects/rdp-classifier/

8 http://www.drive5.com/muscle/

9 https://www.mzcloud.org/

10 http://www.chemspider.com/

11 https://www.metaboanalyst.ca/
