# Supplementary material for: Effect of Fermented Corn-Soybean Meal on Serum Immunity, the Expression of Genes Related to Gut Immunity, Gut Microbiota, and Bacterial Metabolites in Grower-Finisher Pigs
Source: Front Microbiol. 2019 Nov 20;10:2620. doi: 10.3389/fmicb.2019.02620 (PMC6879430; doi:10.3389/fmicb.2019.02620)
Supplement: Supplementary file 1 [file Data_Sheet_1.docx]

Supplementary Information for:

**Effect of Fermented Corn-soybean Meal on Serum Immunity, the Expression of Genes Related to Gut immunity, Gut Microbiota, and Bacterial Metabolites in Grower-finisher Pigs**

**Junfeng Lu, Xiaoyu Zhang, Yihao Liu, Haigang Cao, Qichun Han, Baocai Xie, Lujie Fan, Xiao Li, Jianhong Hu, Gongshe Yang, Xin′e Shi^*^**

Key Laboratory of Animal Genetics, Breeding and Reproduction of Shaanxi Province, Laboratory of Animal Fat Deposition and Muscle Development, College of Animal Science and Technology, Northwest A&F University, Yangling, China

*** Correspondence:**Xin**′**e Shi
xineshi@nwafu.edu.cn

**This file include:**

Supplementary Table 1-2

Supplementary Figure1-2

Supplementary Data Sheet 2

**Supplementary Tables**

**Table S1.** Primers used for encoding the V4–V5 region of 16S rDNA. ^a^i5 and i7 represent 8nt index sequences that enable the identification of sequences originated from each prespecified DNA sample.

**Table S2.** Serum biochemical parameters. Ctrl: pigs fed with unfermented corn-soybean meal; FF: pigs fed with fermented corn-soybean meal. Data were shown as mean ± SEM (n=5-6). There was no significance between two groups.

**TABLE S1** **|** Primers used for encoding the V4–V5 region of 16S rDNA

| **Direction** | **Primer** |
| --- | --- |
| Forward | 5′-AATGATACGGCGACCACCGAGATCTACAC-i5[^a^](https://www.ncbi.nlm.nih.gov/pmc/articles/PMC5330390/table/T2/#t2n1)TATGGTAATTGTGTGCCAGCMGCCGCGGTAA-3′ |
| Reverse | 5′-CAAGCAGAAGACGGCATACGAGAT-i7[^a^](https://www.ncbi.nlm.nih.gov/pmc/articles/PMC5330390/table/T2/#t2n1)-AGTCAGTCAGGCCCCGTCAATTCMTTTRAGT-3′ |

**TABLE S2 |** Serum biochemical parameters

| **Items** | **Ctrl** | **FF** |
| --- | --- | --- |
| TP (g/L) | 65.84 ± 1.70 | 67.57 ± 1.18 |
| TG (mmol/L) | 0.42 ± 0.02 | 0.38 ± 0.04 |
| GLU (mmol/L) | 4.70 ± 0.36 | 4.30 ± 0.14 |
| LDL-c (mmol/L) | 0.99 ± 0.08 | 0.97 ± 0.06 |
| ALB (g/L) | 27.18 ± 0.18 | 28.40 ± 0.52 |
| GLO (g/L) | 38.66 ± 1.51 | 39.17 ± 0.93 |
| A/G | 0.71 ± 0.04 | 0.73 ± 0.02 |

**Supplementary Figures**

**Figure S1.** 16S RNA bacterial sequences represent in fermented feed samples. Bar graph of average values of relative abundance (percentage of sequences) of the most abundant bacterial groups: phylum(A), class (B), family (C), genus(D) found in the fermented feed (n=3).

**Figure S2.** Rarefaction curves comparing the number of sequences with the number of phylotypes found in the 16S rRNA gene libraries from microbiota in the duodenal digesta and colonic digesta of pigs fed unfermented and fermented feed diets. (A) Ctrl colonic digesta, (B) Ctrl duodenal digesta, (C) FF colonic digesta and (D) FF duodenal digesta(n=6).

**
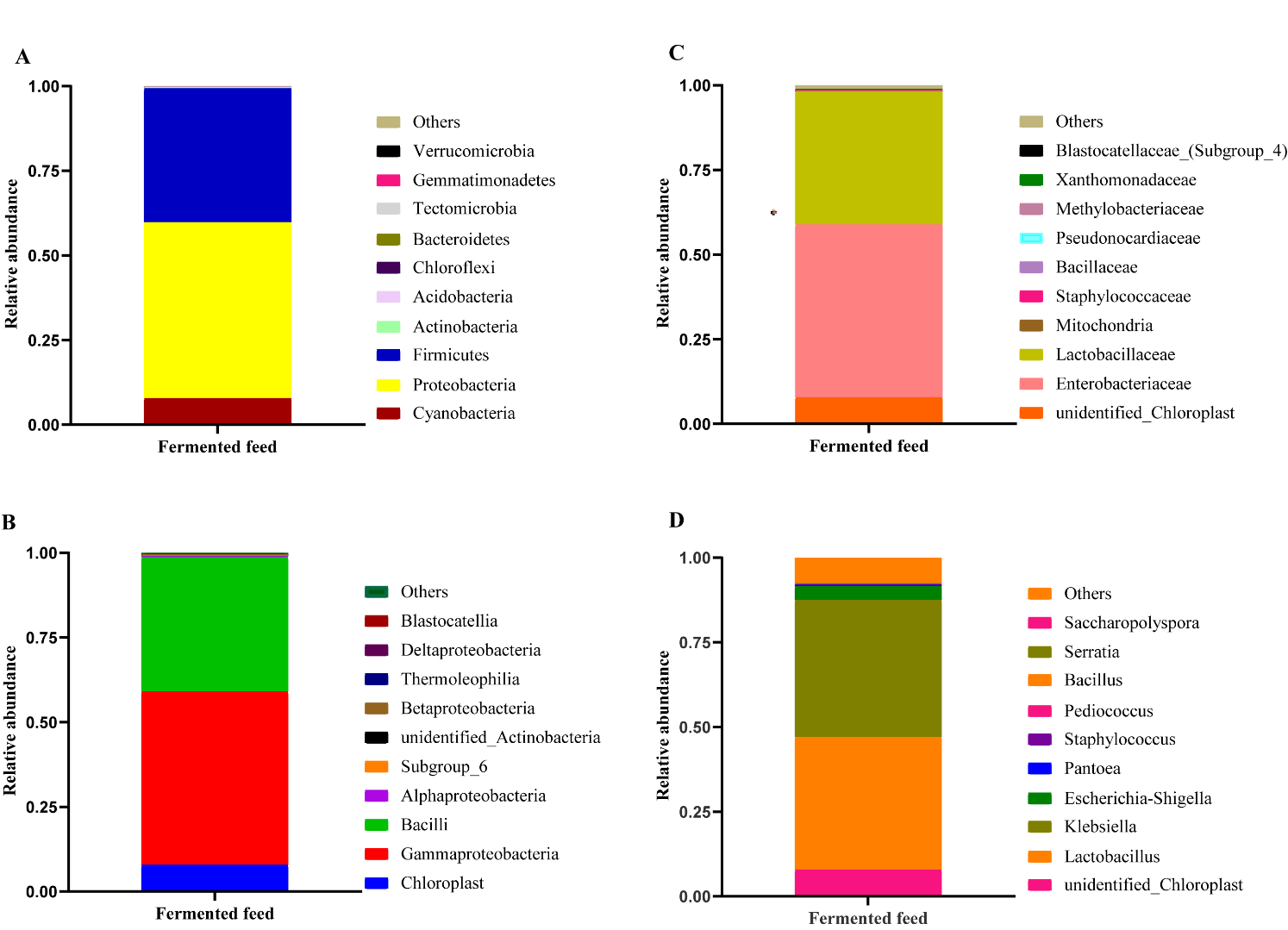
**

**FIGURE S1 |** 16S RNA bacterial sequences represent in fermented feed samples


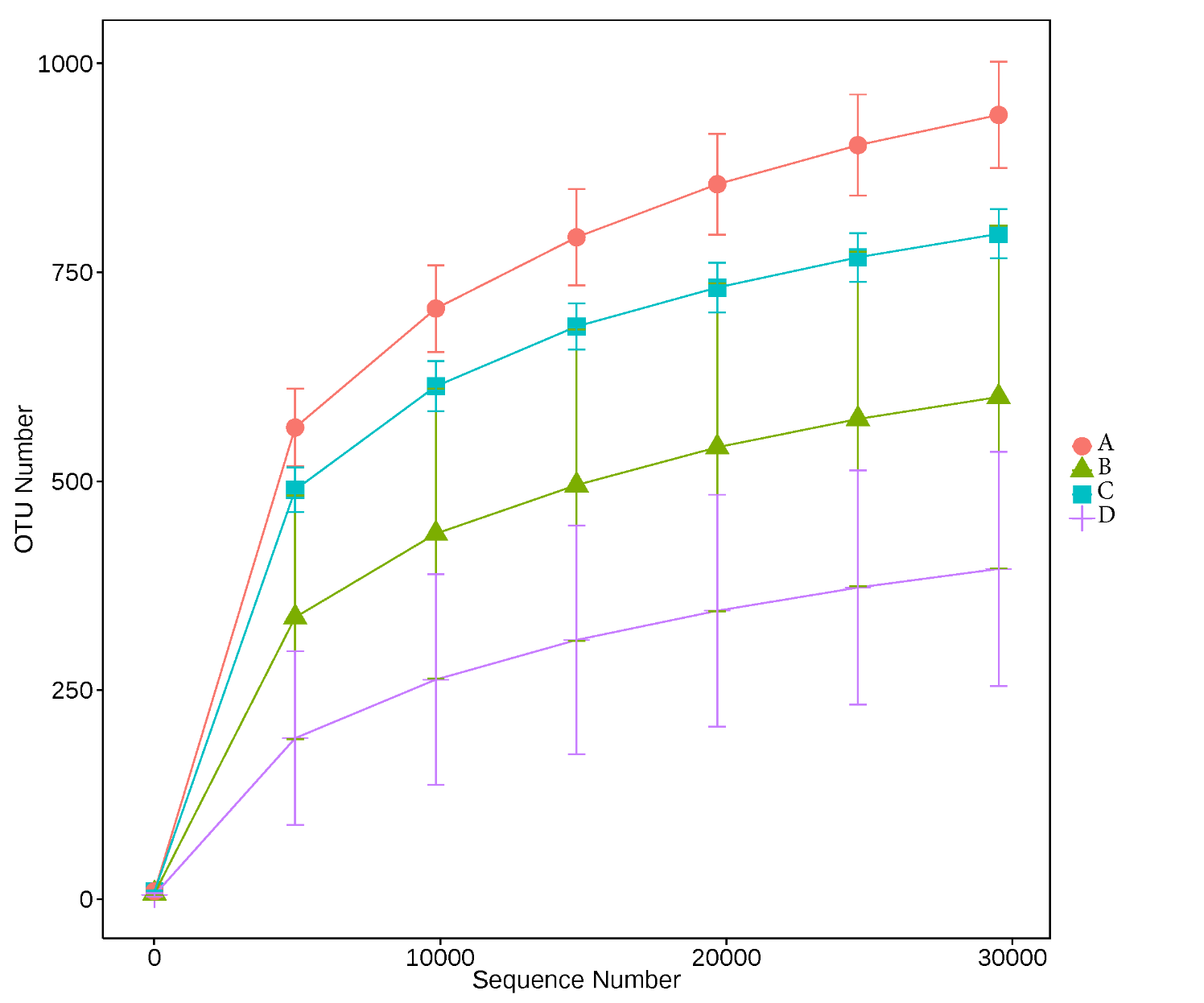


**FIGURE S2 |** Rarefaction curves comparing the number of sequences with the number of phylotypes found in the 16S rRNA gene libraries from microbiota in the duodenal digesta and colonic digesta of pigs fed unfermented and fermented feed diets

**Supplementary Data**

**Data Sheet 2.** Detailed information about the 1,351 biomarker metabolites detected by LC-MS (ESI+) and LC-MS (ESI+). (See separate Excel file)
